# Quorum Sensing Controls the CRISPR and Type VI Secretion Systems in *Aliivibrio wodanis* 06/09/139

**DOI:** 10.3389/fvets.2022.799414

**Published:** 2022-02-08

**Authors:** Amudha Deepalakshmi Maharajan, Erik Hjerde, Hilde Hansen, Nils Peder Willassen

**Affiliations:** ^1^Norwegian Structural Biology Center and Department of Chemistry, Faculty of Science and Technology, UiT The Arctic University of Norway, Tromsø, Norway; ^2^Centre for Bioinformatics, Department of Chemistry, Faculty of Science and Technology, UiT The Arctic University of Norway, Tromsø, Norway

**Keywords:** CRISPR, T6SS, QS, *Aliivibrio wodanis* 06/09/139, LitR and AinS

## Abstract

For bacteria to thrive in an environment with competitors, phages and environmental cues, they use different strategies, including Type VI Secretion Systems (T6SSs) and Clustered Regularly Interspaced Short Palindromic Repeats (CRISPR) to compete for space. Bacteria often use quorum sensing (QS), to coordinate their behavior as the cell density increases. Like other aliivibrios, *Aliivibrio wodanis* 06/09/139 harbors two QS systems, the main LuxS/LuxPQ system and an N-acyl homoserine lactone (AHL)-mediated AinS/AinR system and a master QS regulator, LitR. To explore the QS and survival strategies, we performed genome analysis and gene expression profiling on *A. wodanis* and two QS mutants (Δ*ainS* and Δ*litR*) at two cell densities (OD600 2.0 and 6.0) and temperatures (6 and 12°C). Genome analysis of *A. wodanis* revealed two CRISPR systems, one without a *cas* loci (CRISPR system 1) and a type I-F CRISPR system (CRISPR system 2). Our analysis also identified three main T6SS clusters (T6SS1, T6SS2, and T6SS3) and four auxiliary clusters, as well about 80 potential Type VI secretion effectors (T6SEs). When comparing the wildtype transcriptome data at different cell densities and temperatures, 13–18% of the genes were differentially expressed. The CRISPR system 2 was cell density and temperature-independent, whereas the CRISPR system 1 was temperature-dependent and cell density-independent. The primary and auxiliary clusters of T6SSs were both cell density and temperature-dependent. In the Δ*litR* and Δ*ainS* mutants, several CRISPR and T6SS related genes were differentially expressed. Deletion of *litR* resulted in decreased expression of CRISPR system 1 and increased expression of CRISPR system 2. The T6SS1 and T6SS2 gene clusters were less expressed while the T6SS3 cluster was highly expressed in Δ*litR*. Moreover, in Δ*litR*, the *hcp1* gene was strongly activated at 6°C compared to 12°C. AinS positively affected the *csy* genes in the CRISPR system 2 but did not affect the CRISPR arrays. Although AinS did not significantly affect the expression of T6SSs, the hallmark genes of T6SS (*hcp* and *vgrG*) were AinS-dependent. The work demonstrates that T6SSs and CRISPR systems in *A. wodanis* are QS dependent and may play an essential role in survival in its natural environment.

## Introduction

Quorum sensing (QS) is a cell density-dependent cell-to-cell communication system in which bacteria produce and respond to signaling molecules called autoinducers (AIs), which subsequently activates the QS transcriptional regulator to control specific functions such as bioluminescence, motility, biofilm, secretion and virulence (1–5). Multiple QS systems have been described in several *Vibrio* species (6). Two QS systems, AinS/AinR and LuxS/LuxPQ that are believed to work through phosphorelay mechanism have been identified in the genome of *Aliivibrio wodanis* (7). The AinS/AinR QS system produces AI-1 known as N-acyl homoserine lactones (AHLs) and is present in many Gram-negative bacteria (2, 8). These AHL-mediated QS systems are used for intra-species communication (8, 9). The LuxS/LuxPQ QS system is present in a wide variety of Gram-negative and Gram-positive bacteria, and produces the AI-2 called furanosyl borate diester and is involved in inter-species communication (10, 11). These two QS systems are known to work in parallel in *Vibrio harveyi, Aliivibrio fischeri* and *Vibrio cholerae* (12–14). At low cell density, when the AI concentrations are low, the AI receptors act as kinases and relay phosphate to the RpoN (σ54)-dependent activator LuxO via phosphotransferase LuxU. This, in turn, activates the expression of *qrr* sRNAs, which together with RNA chaperone Hfq represses translation of the mRNA encoding the master regulator *LitR* (9, 14, 15). At high cell density, the signaling molecules reach a threshold concentration and bind to the receptors to dephosphorylate LuxO, which terminates the *qrr* sRNA transcription. In the absence of Qrr sRNA, *litR* is activated to regulate hundreds of genes (1, 14, 16).

In the environment, bacteria co-exist in communities with multiple competitors, including other bacterial species and phages, and have to respond to various cues such as changes in temperature, nutrient and iron availability, pH, osmolarity and salinity (17–22). Hence, bacteria have developed various strategies such as protein secretion, contact-dependent growth inhibition, bacteriocin production and antibiotic production to survive and thrive (23–27). Some strategies are not necessarily harmful to competitors, such as adhesion, exopolysaccharide production, siderophore production, motility, biofilm formation, heat shock response and quorum quenching (28–34). Other strategies developed, such as defense mechanisms against phages or mobile genetic elements (MGEs), including restriction-modification, receptor modification and clustered regularly interspaced short palindromic repeats-CRISPR associated (CRISPR-Cas) are for protection (35–37).

The CRISPR-Cas system is an adaptive immune system against invading nucleic acids from phages and other MGEs and is composed of *cas* genes, a leader sequence and a CRISPR array with repeats separated by several spacer sequences (35, 38). Two CRISPR classes have been identified with six main types and several subtypes, which are categorized based on the types of *cas* genes, direct repeats and gene arrangement where class 1 includes the type I, III and IV whereas type II, V and VI belong to class 2 (39–41). Several *Vibrio* species harbor the type I CRISPR system classified into subtypes such as type I-A, I-B, I-C, I-D, I-E and I-F (40, 42, 43). QS regulation of CRISPR system has been described in bacteria like *Pseudomonas aeruginosa, Serratia* sp. and *Chromobacterium violaceum* (44–47).

QS regulates type VI secretion systems (T6SSs) in several vibrios such as *V. cholerae, Vibrio parahaemolyticus, Vibrio anguillarum, Vibrio fluvialis* and *Vibrio alginolyticus* (48–52). T6SS is one of the largest contact-dependent secretion system bacteria use to transport T6SS effectors (T6SEs) into eukaryotic hosts, bacterial competitors or the environment (53–56). In some bacteria, T6SSs are also known to be involved in the uptake of metal ion (57, 58). The T6SS was first identified in *V. cholerae* as a virulence-associated secretion (*vas*) gene cluster and later in many other bacteria (59–61). The T6SEs are toxin molecules with anti-bacterial or anti-eukaryotic activity (62–64). Several anti-bacterial effector molecules such as amidases, glycoside hydrolases, lipases, phospholipases and nucleases and anti-eukaryotic effectors such as VasX, the Multifunctional-autoprocessing repeats-in-toxin and EvpP have been identified (65–69). T6SS gene clusters often encode immunity proteins close to the effector genes in order to neutralize their effector molecules to prevent self or sibling-killing (70). For instance, immunity proteins such as antitoxin TsaB in *V. cholerae* have been reported to protect self-killing against effectors VgrG-3 and Tse2, respectively (71). In addition, immunity protein-independent mechanisms like envelope stress response and two-component systems can facilitate self-protection (72).

*A. wodanis* is a Gram-negative, rod-shaped, non-luminescent and motile bacterium with multiple polar flagella (73). *A. wodanis* strains grow in the range of temperatures and salt concentrations between 4–25°C and 1–4% respectively (73). The genome of *A. wodanis* 06/09/139 contains two chromosomes and 4 plasmids (7). *A. wodanis* has been repeatedly isolated together with *Moritella viscosa (M. viscosa)* from Atlantic salmon (*Salmo salar*) during outbreaks of winter ulcer that mainly occurs at a temperature below 8°C (74, 75). Infected fish can survive when the temperature rises above 8°C (74, 76). Winter ulcer causes mortality and significant losses in farming industry and is characterized by large ulcers, hemorrhages and internal tissue necrosis in the infected fish (74, 77). Although *M. viscosa* is the primary agent for the disease, the role of *A. wodanis* and the mechanism behind the co-existence with *A. wodanis* in the winter ulcers are still unclear (74, 77). Experimental study reproducing field observation reveal that *A. wodanis* affects the progression of *M. viscosa* infection and is responsible for the chronic pathogenesis in fish (78). *A. wodanis* adheres to Atlantic salmon head kidney cells and in a bath challenge, *A. wodanis* separately produces clinical symptoms such as fin rot and other internal pathological symptoms in Atlantic salmon and it can co-infect Atlantic salmon together with *M. viscosa* (78). In a co-cultivation experiment, *A. wodanis* impedes the growth of *M. viscosa* and when both the bacteria were implanted together in fish abdomen, *A. wodanis* alters the gene expression of *M. viscosa* (7). It has been further hypothesized that *A. wodanis* perhaps uses bacteriocin to impede the growth and virulence of *M. viscosa* (7). In our previous studies we have reported that *A. wodanis* produces one AHL and encodes two QS systems (7, 79). In the cell culture studies, *A. wodanis* is known to be cytotoxic to different salmon cell lines when treated with supernatants harvested at cell densities higher than OD_600_ (optical density measured at 600 nm) of 6.0 (78, 80). Moreover, in a HPLC-MS/MS analysis, the AHL production in *A. wodanis* begins at the early log phase and increases with increase in cell density along the growth curve (80). Hence in this transcriptomics study, we chose two cell densities one at the early log phase (OD_600_ 2.0) and the other at the cell density close to the end log phase (OD_600_ 6.0) to study the role of cell densities in gene expression. Furthermore, in our recent study, we found that the temperature 6°C which is lower than the winter ulcer disease threshold temperature 8°C, has more impact on AHL production and cytotoxicity in CHSE cell line than 12°C (80). Therefore, in this study, we wanted to analyze the effects of 6°C and 12°C in gene expression. We have also shown that *A. wodanis* uses the QS to regulate growth, motility, siderophore- and protease production, hemolysis as well as cytotoxicity in the Chinook salmon embryo (CHSE) cell line (80). Considering the importance of understanding the QS and survival strategies in *A. wodanis*, we performed genome analysis and RNA sequencing (RNA-Seq) to reveal the global gene expression in the wild type and its QS mutant strains Δ*ainS* and Δ*litR*.

## Materials and Methods

### Bacterial Strains and Growth Conditions

The *A. wodanis* 06/09/139 used was originally isolated from the head kidney of an Atlantic salmon on the west coast of Norway in 2006 (78). The construction of the Δ*ainS* and Δ*litR* in-frame mutants by allelic exchange has been described in a recent study (80). *A. wodanis* 06/09/139 and the mutants from glycerol stocks were grown at 12°C for 3 days on Luria-Bertani Agar (Difco BD Diagnostics Sparks, MD, USA) with a total concentration of 1.0% (wt/vol) peptone (Sigma-Aldrich, St. Louis, MO, USA), 0.5% (wt/vol) yeast extract (Merck, Darmstadt, Germany), 2.5% NaCl (wt/vol) (Sigma-Aldrich, St. Louis, MO, USA) and 1.5% agar (Sigma-Aldrich, St. Louis, MO, USA). The pH of the media was adjusted to 7.5. Three biological replicates of pre-cultures of *A. wodanis* 06/09/139 and the mutants Δ*ainS* and Δ*litR* were grown from a single colony in 2 ml of Luria-Bertani Broth (LB2.5) overnight at 12°C, 220 rpm.

### RNA Extraction and rRNA Depletion

Pre-culture biological triplicates of *A. wodanis* 06/09/139, Δ*ainS* and Δ*litR* were diluted to a start OD_600_ of 0.01 in LB2.5 in a final volume of 60 ml using a 250 ml baffled flasks. The cultures were grown further in parallel using two Infors HT Multitron incubators set at 6 and 12°C at 220 rpm. The cultures were diluted 1:10 for OD_600_ measurement and harvested at low cell density (LCD) OD_600_ of 2.0 and high cell density (HCD) OD_600_ of 6.0. A small culture volume (1 ml) was harvested and mixed with two volumes of RNAprotect Bacteria reagent (Qiagen, Hilden, Germany). The treated cultures were then incubated for 5 min at room temperature and vortexed for a few seconds before centrifuging at 13,000 rpm for 2 min in a cold Heraeus fresco 21 centrifuge (Thermo Scientific, Waltham, MA, USA). The pellets were flash-frozen with liquid nitrogen and stored at −80°C until RNA isolation. The total RNA from the cell pellets was isolated using the Masterpure^TM^ complete DNA/RNA purification kit (Epicenter, Madison, WI, USA) according to the manufacturer's instructions. The RNA concentration was measured in NanoDrop^TM^ 2000c spectrophotometer (Thermo Scientific, Waltham, MA, USA). Ribosomal RNA (rRNA) was depleted from the total RNA using a Ribo-zero rRNA removal kit for bacteria (Illumina, San Diego, CA, USA). The RNA quality before and after rRNA depletion was determined using Agilent 2100 Bioanalyzer (Agilent Technologies, Santa Clara, CA, USA).

### RNA Sequencing and Data Analysis

The mRNA libraries were prepared using the TrueSeq stranded mRNA library kit (Illumina, San Diego, CA, USA) and sequenced on Nextseq 500 (Illumina, USA) using 150 cycles mid-output kit and run as a 75 bp paired-end reads at Norwegian Sequencing Center. The image analysis and base calling were performed using Illumina's RTA software version 2.4.6. Reads with low base call quality were removed using Illumina's default chastity criteria. The quality of the raw sequencing data was controlled using FastQC version 0.11.5 (https://www.bioinformatics.babraham.ac.uk/projects/fastqc/). The gene expression levels were determined using EDGE-pro v1.0.1 (81) and DESeq2 (82) with default parameters. EDGE-pro was used to align the reads to the reference genome (GCA_000953695.1) and convert the raw coverage into reads per kilobase of gene per million reads mapped (RPKM). DEseq2 was used to estimate the comparative differential gene expression values and provide the output as a log2fold change value with *p-value*. The log2fold change values were converted into fold change (FC) values, and the cutoff values of ≥ 2.0 or ≤ −2.0 with *p*adj values of < 0.05 were counted as significantly differentially expressed genes (DEGs). The transcriptome profiles of each strain was compared as high cell density against low cell density (tpHCD/LCD), high growth temperature 12°C against low temperature 6°C (tp12°C/6°C) and mutants against wild type (tpΔ*litR*/WT and tpΔ*ainS*/WT). These abbreviations of comparisons are used throughout this paper. The RNA sequence data of wild type *A. wodanis*, Δ*ainS* and Δ*litR* have been deposited in the European Nucleotide Archive (www.ebi.ac.uk/ena) under study accession number PRJEB34433.

### Functional Gene Family and Pathway Analysis

KEGG BRITE and KEGG pathway mapper were used to map the DEGs (FC values ≥ 2.0 or ≤−2.0) found when wild type was compared against cell densities (tpHCD/LCD) and temperatures (tp12°C/6°C) and also when mutants were compared against wild type (tpΔ*litR*/WT and tpΔ*ainS*/WT) at different cell densities and temperatures against the reference organism *A. wodanis* 06/09/139 (83).

### Identification of CRISPR Systems, Protospacers and Prophage

The CRISPR-Cas operon in *A. wodanis* 06/09/139 genome was identified using CRISPRCasFinder (84), and a homology search was performed using KEGG Sequence Similarity Database (SSDB) (83). An intergenic distance of < 100 bp between genes was considered as a single operon in this study. The CRISPR arrays with direct repeats and spacers were identified from the genome of *A. wodanis* using the CRISPR finder tool (85), while the protospacers were identified using CRISPR target (86). The prophages in *A. wodanis* were identified using the phage search tool (PHASTER) (87, 88).

### Identification of Type VI Secretion Systems and Type VI Secretion Effectors

The T6SS gene clusters in *A. wodanis* 06/09/139 were identified using SecReT6 (89) and a homology search against other T6SS gene clusters using KEGG SSDB database (83). The naming of T6SS genes of *A. wodanis* 06/09/139 in this study follows the naming of *V. cholerae* T6SS (90). Potential T6SEs in *A. wodanis* were predicted using the Bastion 6 tool (91).

## Results

We have previously shown that *A. wodanis* 06/09/139 produces one AHL (3OHC10-HSL) and encodes genes for QS (7, 79). Moreover, in a recent study, the QS system in *A. wodanis* 06/09/139 was found to affect various phenotypes that are possibly linked to the winter ulcer disease development (80). Genome analysis and transcriptome profiling was therefore performed to further study the QS and survival strategies in *A. wodanis* by comparing wild type and its Δ*litR* and Δ*ainS* mutants at low (OD_600_ of 2.0) and high cell density (OD_600_ of 6.0) and two different temperatures, 6 and 12°C.

### Differential Expressed Genes

The transcriptome profiles of *A. wodanis* and mutant strains Δ*litR* and Δ*ainS* at different cell densities and temperatures are listed in Supplementary Table 1. The average of mapped reads to the reference genome of *A. wodanis* was above 90% in all samples suggesting that the transcriptome data were sufficient for further analysis.

**Figure 1:**
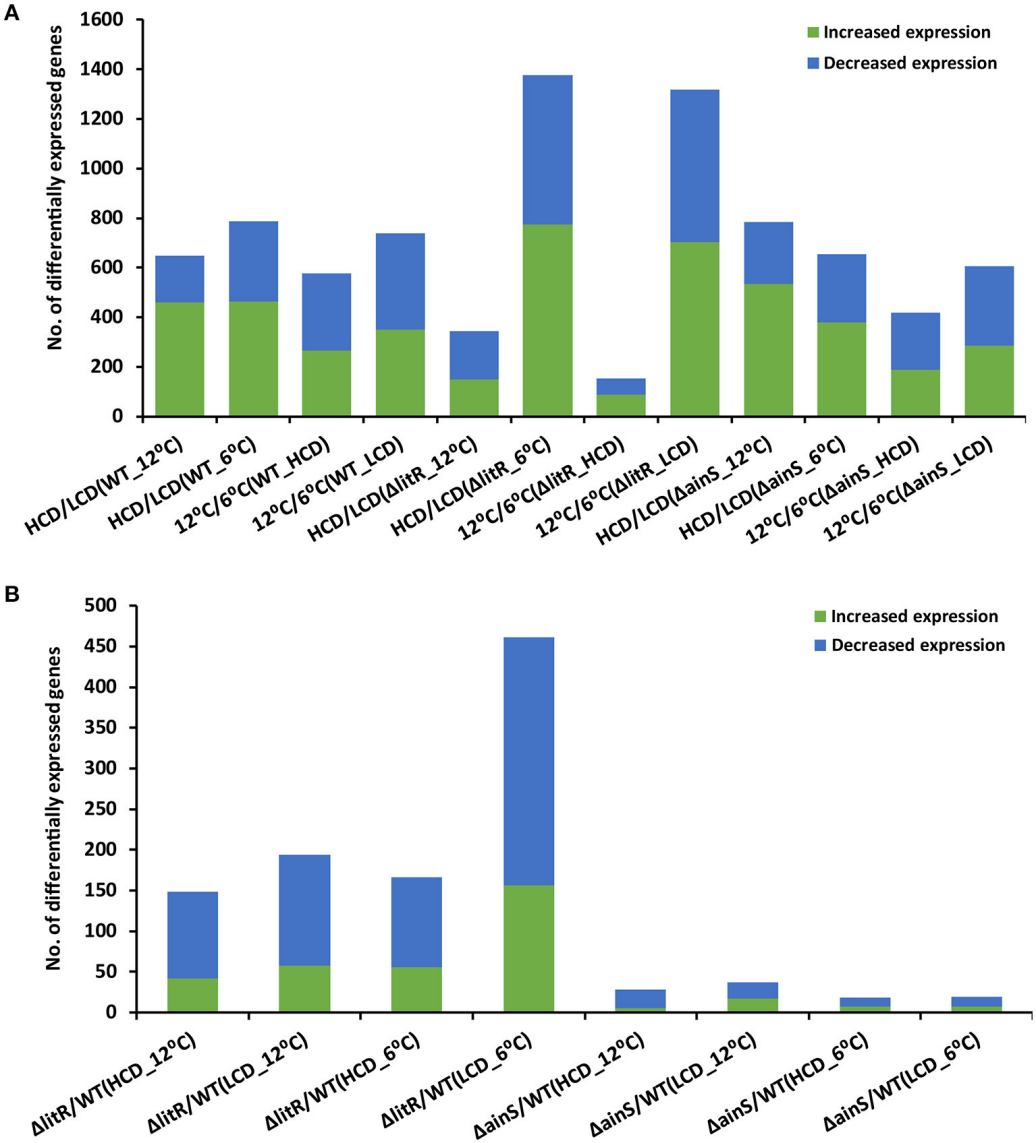
The total number of differentially expressed genes in *A. wodanis* 06/09/139, Δ*ainS* and Δ*litR*. **(A)** The bar chart shows the number of increased (green) and decreased (blue) expression of genes when comparing high cell density (HCD) to low cell density (LCD) and growth temperature at 12 to 6°C. **(B)** The bar chart shows the number of increased and downregulated genes in comparisons Δ*litR*/WT and Δ*ainS*/WT.

The total number of DEGs at all tested conditions are shown in Figure 1, and the complete lists of all DEGs are given in Supplementary Table 2. When comparing the transcriptome profiles at HCD relative to LCD (tpHCD/LCD) of the wild type after growth at 6°C, 18% of the total genes (*n* = 4,282) of *A. wodanis* were differentially expressed. Increasing the growth temperature to 12°C lowered this number of DEGs to 15%. Among the tested tpHCD/LCD conditions of wildtype, Δ*litR* and Δ*ainS* (Figure 1A), the highest numbers of DEGs were observed for the Δ*litR* mutant at 6°C, where the number of DEGs account for about 32% of the total genes in of *A. wodanis*. On the other side, the lowest number of DEGs (8% of the total 4282 genes) was observed in the Δ*litR* mutant tpHCD/LCD at 12°C. The *ainS* mutant tpHCD/LCD at 6°C and 12°C showed 15 and 18% DEGs, respectively. Further, the DEGs in tpHCD/LCD (WT_12°C) and tpHCD/LCD (WT_6°C) were sorted using KEGG BRITE and KEGG pathway mapper using *A. wodanis* 06/09/139 as a reference organism. The functional families with at least 15 DEGs were *enzymes, transporters, secretion system, bacterial motility proteins* and *ribosome* as shown in Supplementary Figure 1A. In addition, in the wild type tpHCD/LCD comparison, at 6°C, a few DEGs (*n* = 5) with FC values ≥ 2.0 were also mapped into the functional families *prokaryotic defense system*, which includes the CRISPR related genes. The KEGG pathway mapping revealed that the DEGs (*n* ≥ 30) in the wild type tpHCD/LCD were involved in *metabolic pathways, microbial metabolism in diverse environments, biosynthesis of secondary metabolites, ABC transporters, biosynthesis of antibiotics, two-component system, carbon metabolism* and the *ribosome*. The numbers of DEGs involved in these pathways were similar in wild type tpHCD/LCD at both temperatures except in the *ribosome* pathway. At tpHCD/LCD (6°C), 42 DEGs mapped into *the ribosome* pathway, whereas only one DEG mapped at tpHCD/LCD (12°C) (Supplementary Figure 2A).

Comparison of the transcriptome profiles of the wild type grown at 12°C and 6°C (tp12°C/6°C) revealed DEGs, accounting for 13 and 17% of the total 4282 *A. wodanis* genes at HCD and LCD, respectively (Figure 1A). In the Δ*ainS* mutant (tp12°C/6°C), 10 and 14% of total genes were differentially expressed at HCD and LCD, respectively. Compared to the wild type and the Δ*ainS* mutant, 31% of the total genes in the Δ*litR* mutant were differentially expressed (tp12°C/6°C) at LCD, whereas at HCD, 4% of the total genes were differentially expressed. Further, the DEGs in wild type (tp12°C/6°C) with FC values ≥ 2.0 and ≤−2.0 were sorted into KEGG BRITE and KEGG pathway mapper. The functional families with DEGs of ≥ 15 were *enzymes, transporters, ribosome, ribosome biogenesis* and *non-coding RNAs* (Supplementary Figure 1B). Few DEGs (*n* < 10) in wild type (tp12°C/6°C) mapped into families such as *secretion system* and *prokaryotic defense system*. The KEGG pathway mapping revealed that the DEGs (*n* ≥ 30) in the WT(tp12°C/6°C) were involved in pathways such as *metabolic pathways, microbial metabolism in diverse environments, biosynthesis of secondary metabolites, ABC transporters, biosynthesis of antibiotics, two-component system, carbon metabolism, purine metabolism, Aminoacyl-tRNA biosynthesis* and *ribosome*. The numbers of DEGs involved in these pathways were higher at tp12°C/6°C (LCD) than at tp12°C/6°C (HCD). For example, 43 DEGs at LCD mapped to the *ribosome pathway* while only 6 DEGs mapped at HCD (Supplementary Figure 2B).

When comparing the profile of the Δ*litR* mutant to the wild type tpΔ*litR*/WT at HCD, 3 and 4% of the total genes were differentially expressed at 12 and 6°C, respectively. In the tpΔ*litR*/WT (HCD_6°C), 4% of the total genes were differentially expressed. In tpΔ*litR*/WT (LCD_12°C), 5% of total genes whereas in tpΔ*litR*/WT (LCD_ 6°C), 11% of the total genes were found to be differentially expressed. The number of DEGs was two times higher at 6°C and LCD compared to 12°C and HCD (Figure 1A). When comparing the expression profile of the Δ*ainS* mutant with the wild type (tpΔ*ainS*/WT) at HCD, 0.7 and 0.4% of the total genes were differentially expressed at 12 and 6°C, respectively. In tpΔ*ainS*/WT, during LCD and 12°C, 0.9% of the total genes and at LCD and 6°C, 0.4% of the total genes were found to be differentially expressed. The DEGs in Δ*litR* and Δ*ainS* mutants were sorted using KEGG BRITE (Supplementary Figure 3A) and mapped into KEGG reference pathway for *A. wodanis* 06/09/139 (Supplementary Figure 4A). The DEGs (*n* ≥ 5) in the Δ*litR* and Δ*ainS* mutants compared to wild type with FC values of ≥ 2.0 and ≤−2.0 were associated with functional families such as *enzymes, secretion system, bacterial motility proteins, transporters* and *prokaryotic defense system*. The KEGG pathway mapping revealed that the DEGs in the tpΔ*litR*/WT and tpΔ*ainS*/WT mutants affected several pathways, including *metabolic pathways, two-component system, biosynthesis of secondary metabolites, Quorum sensing* and *bacterial chemotaxis* (Supplementary Figures 3B, 4B). Together our results suggest that, LitR is a crucial global regulator of genes at 6°C and at low cell density. As well, LitR has more impact on gene expression than AinS.

### Key QS Genes of *A. wodanis* are Cell Density and Temperature Dependent

Only a few genes known to be involved in the *A. wodanis* QS system were differentially expressed under the experimental conditions tested (Supplementary Table 3). When comparing the wild type transcriptome profile at HCD to LCD (tpHCD/LCD), the AI synthase gene *ainS* (AWOD_I_1040) and master QS regulator gene *litR* (AWOD_I_0419) showed FC values of 2.01 and 2.24, at 6°C, respectively, while no significant differences were observed at 12°C.

When the wild type profile at high temperature was compared at low temperature (tp12°C/6°C), the *qrr* sRNA gene (AWOD_I_sRNA_054) was significantly higher with FC value of 2.92 at LCD, whereas no difference in its expression was observed at HCD. Temperature alone did not significantly impact other genes of the QS system at either HCD or LCD (Supplementary Table 3).

Comparing the profile of the Δ*litR* mutant relative to the wild type (tpΔ*litR*/WT), only the FC value of *ainS* at LCD and 12°C was significant (FC = −1.93, *p*_*adj*_ value < 0.05), although slightly lower than the cutoff FC value used in this study. The deletion of *litR* did not affect other QS related genes in *A. wodanis* under the conditions tested. On the other hand, when comparing the Δ*ainS* mutant profile to the wildtype (tpΔ*ainS*/WT), the *litR* expression was significantly lower at all conditions examined. The FC values of *litR* were notably lower at HCD compared to LCD. At HCD, the FC values for *litR* at 6°C and 12°C were −2.53 and −2.87, respectively. At LCD, the FC values at 6°C and 12°C were −1.93 and −2.04, respectively, indicating that the expression of *litR* in the Δ*ainS* mutant increases with increased density and independent of the temperature. Furthermore, the phosphorelay protein-encoding gene *luxU* (AWOD_I_0921) expression was significantly higher in the Δ*ainS* mutant at HCD (FC = 2.81) and LCD (FC = 2.17) at 6°C, whereas at 12°C no differences were observed. Hence, the *luxU* expression in the Δ*ainS* mutant was slightly higher at HCD compared to LCD and its expression was affected only at low temperature.

### Differential Expression of CRISPR-Cas System in *A. wodanis*

**Figure 2:**
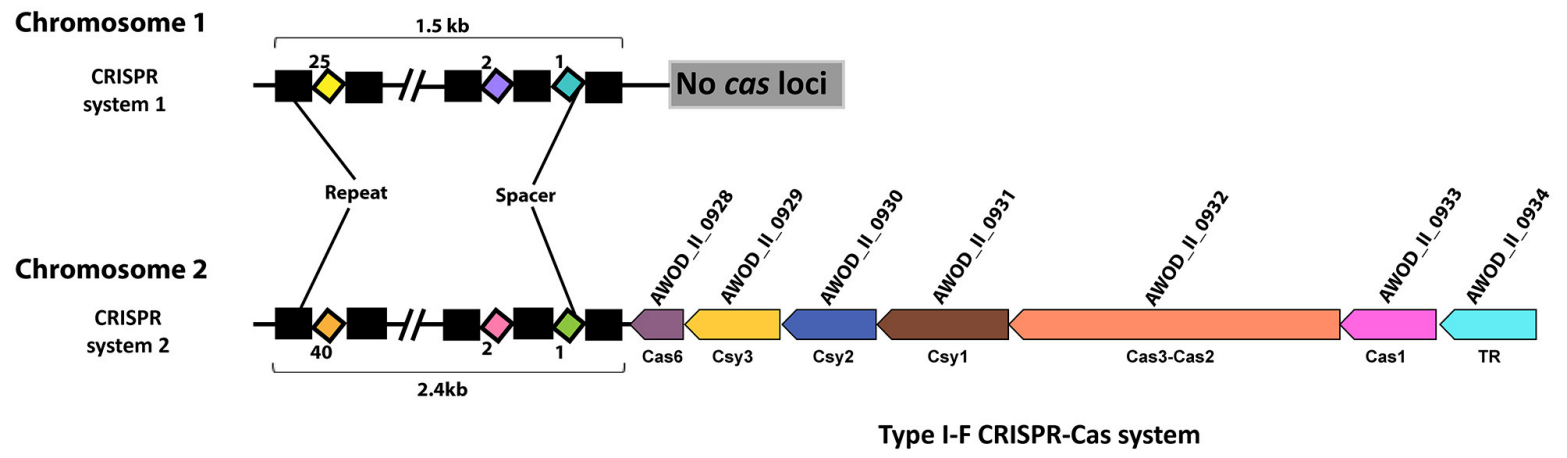
CRISPR systems in *A. wodanis* 06/09/139. The figure shows the CRISPR arrays with repeats (black rectangle) and spacers (colored diamonds). In CRISPR system 2, the CRISPR array is located close to the *cas* operon (arrows) of the Type I-F CRISPR-Cas system. TR denotes transcriptional regulator.

We identified two CRISPR systems in the genome of *A. wodanis* using CRISPRCas finder (84), where CRISPR system 1, located on chromosome 1, did not contain any *cas* loci, whereas CRISPR system 2, located in chromosome 2, contains the *cas* loci (Figure 2). The CRISPR system 2 was classified as a Type I-F CRISPR system. A total number of 25 and 40 spacers with a length of 32-33 nucleotides were identified in CRISPR system 1 and 2, respectively (Supplementary File S4). When the spacers were searched against the phage databases using CRISPR Target, spacers matched to the protospacers with at least 5 single nucleotide polymorphisms. For example, spacer 11 of CRISPR system 1 matched to the *Vibrio* phage CTX plasmid pCTX-2, whereas spacer 19 of CRISPR system 2 matched to the *Staphylococcus* phage phiSP38-1 with a score of 20.

The data shows that in the wild type, the *csy3, cas1*, and *cas3* genes of CRISPR system 2 have significantly higher FC values (FC > 2.0) at HCD than at LCD after growth at 6°C. No significant differences in FC values of the *cas* genes were observed in wild type (tpHCD/LCD) at 12°C (Supplementary Table 5). Cell density had no impact on the expression of CRISPR arrays of CRISPR systems 1 and 2 at either temperature.

When the expression profile at 12°C was compared to 6°C, at LCD and HCD, the CRISPR arrays of system 1 displayed significantly lower FC values (FC value = ~−2.0), suggesting temperature difference influences CRISPR system 1. However, temperature had no effect on the CRISPR system 2.

Further, the expression data showed that the expression of *cas* genes of CRISPR system 2 were strongly reduced in the Δ*litR* mutant at all conditions examined. The FC values of the *cas* genes in the Δ*litR* mutant increased with increasing cell density at both temperatures. Notably, in the Δ*litR* mutant at 6°C, the FC values of *csy3* and *csy1* were twice as high at HCD (FC = − approx. 10.0) compared to LCD (FC = − approx. 5.0). At LCD, the genes *csy1, csy2, csy3*, and *csy4* had lower FC values at 6°C compared to 12°C, whereas the *cas1* and *cas3* showed higher FC values (Supplementary Table 5). However, at HCD, the highest FC values for *cas* genes were observed at 6°C than at 12°C. The CRISPR array of system 2 in the Δ*litR* mutant showed significantly lower expression at LCD and 12°C (FC value = −1.96) and LCD and 6°C (FC value = −2.69). However, at HCD, the expression of the CRISPR array of system 2 was not significantly different. The data also showed that the CRISPR array of system 1 was highly expressed at HCD and 12°C, whereas the FC values were not significantly different at HCD and 6°C or at LCD at 6°C and 12°C. In the Δ*ainS* mutant compared to wild type, genes such as *csy2, csy3*, and *csy4* showed significantly lower expression at two experimental conditions, LCD, 12°C and HCD, 6°C. No significant differences were observed in expressions of the CRISPR array of system 1 and 2 in *the* Δ*ainS* mutant.

### *A. wodanis* Harbors Three Main T6SSs and Four Auxiliary Clusters

**Figure 3:**
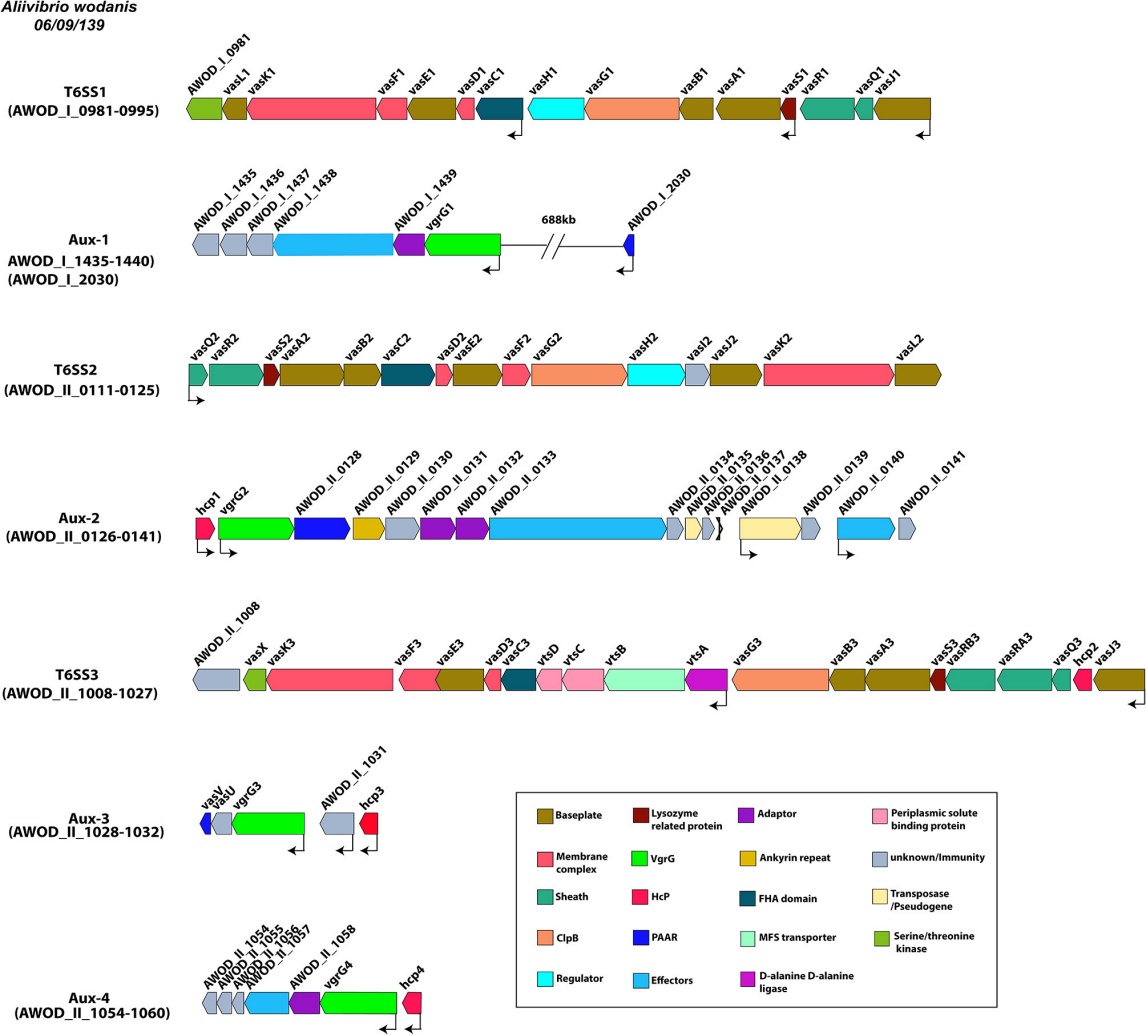
Type VI secretion systems in *A. wodanis* 06/09/139. Graphical representation of the three main T6SSs of *A. wodanis* and the four auxiliary T6SS clusters. The thin black arrows indicate the direction and start of the transcription sites.

*A. wodanis* was found to harbor three T6SSs, each encoding the conserved core and accessory T6SS genes. T6SS1 was located on chromosome 1, whereas the T6SS2 and T6SS3 were on chromosome 2. The T6SS1 gene clusters (AWOD_I_0981-0995) were composed of 3 operons with 15 consecutive genes, whereas the T6SS2 gene clusters consisted of 1 operon with 15 consecutive genes (AWOD_II_0111-0125) and T6SS3 gene clusters with 2 operons with 20 consecutive genes (AWOD_II_1008-1027) as shown in Figure 3. The main T6SS clusters comprised the core components *vasABDEFGHJKLQRS*, valine-glycine repeat protein G (*vgrG*) and Hemolysin Coregulated protein (*hcp*) and the accessory genes *vasCIUVX* (60, 92, 93). The genes *vasABEJL* and *vasDFK* may form the base-plate and membrane complex, respectively (94–96). The genes *vasRQ* and *vasC* may form the outer sheath and the FHA domain, respectively, while *vasG* is believed to act as a chaperone (97, 98). The gene *vasH* may serve as a sigma-54 dependent transcriptional regulator and *vasIS* as lysozyme-related proteins (99). The T6SS3 in *A. wodanis* does not encode the transcriptional regulator *vasH* (99). Four *vgrG* paralogous genes were identified in *A. wodanis*, and using them as markers, four auxiliary clusters were predicted, such as Aux-1 (AWOD_I_1435-1440, AWOD_I_2030), Aux-2 (AWOD_II_0126-0141), Aux-3 (AWOD_II_1028-1032) and Aux-4 (AWOD_II_1054-1060). The auxiliary clusters were located together with the core T6SS genes encoding Proline-alanine-alanine-arginine repeats (PAAR), VgrG and Hcp. The Aux-1 cluster does not encode Hcp, and Aux-4 does not encode a PAAR protein. Four genes encoding adaptor proteins of the DUF4123 family (unknown function) were identified adjacent to *vgrG1, vgrG2* and *vgrG4*, whereas no adaptor protein-encoding gene was present adjacent to *vgrG3*.

### Differential Expression of T6SSs and Auxiliary Clusters of *A. wodanis*

In the wild type, at HCD, the main clusters T6SS1 (*vasR1Q1J1*) and T6SS2 (*vasH1I1J1K1L1*) were highly expressed at both 6 and 12°C, compared to LCD. No differential expression was observed for the T6SS3 (Supplementary Table 6). Furthermore, the complete auxiliary cluster Aux-1 showed higher expression at HCD at both temperatures. The Aux-2, Aux-3 and Aux-4 showed only significant differences in some genes such as AWOD_II_0126 - 0139, AWOD_II_1031 - 1032 and AWOD_II_1054 - 1059, respectively.

The temperature did not affect the expression of the T6SS1 and T6SS3 in the wild type. However, a few genes (*vasQ2, vasR2, vasS2, and vasD2*) of T6SS2 were differentially expressed at HCD, while at LCD, only *vasQ2* was differentially expressed. As for the auxiliary clusters, only the genes encoding membrane protein and DUF4123 (AWOD_I_1435 and AWOD_I_1439) of Aux-1, *hcp1* of Aux-2 and a putative uncharacterized protein (AWOD_II_1031) of Aux-3 were differentially expressed.

When the Δ*litR* mutant was compared to wild type (tpΔ*litR*/WT), the data revealed that the *vasR1Q1J1* operon of T6SS1 was differentially expressed at all tested conditions. The FC values of genes *vasR1Q1J1* were marginally higher at HCD compared to LCD. Particularly the FC values of *vasQ1* gene in Δ*litR* mutant were twice as high at HCD compared to LCD at both temperatures. The FC values of *vasR1Q1J1* operon in the Δ*litR* mutant were significantly higher at 6°C compared to 12°C at both cell densities (Supplementary Table 6). The genes *vasF1D1C1* showed higher FC values in Δ*litR* mutant at LCD than HCD. At 6°C, the complete T6SS2 cluster was differentially expressed in the Δ*litR* mutant at both HCD and LCD. At 12°C, differential expression was only observed in some of the genes in the T6SS2 cluster. Moreover, the FC values of T6SS2 genes were higher at low (6°C) compared to high (12°C) temperature. The T6SS3 gene cluster was only differentially expressed at LCD and 6°C with an average FC value of 2.0. In Δ*litR* mutant, some genes of Aux-1, Aux-2 and Aux-3 were differentially expressed, whereas the Aux-4 showed no differential expression (Supplementary Table 6). In Aux-1, the expression of *vgrG1* was lower in Δ*litR* mutant with an FC value of−4.90 at LCD and 6°C. The genes encoding Hcp1, VgrG2, PAAR motif-containing protein, a protein with ankyrin repeats and the rearrangement hotspot (RHS) protein in the Aux-2 were differentially expressed, where the FC values (FC = −22.75) of *hcp1* at HCD and 6°C was approximately thrice compared to LCD (FC = −8.32). Similarly, three times higher expression of *hcp1* was observed at 6°C compared to 12°C at both HCD and LCD. About 6 of the 16 total genes in the Aux-2 cluster showed significantly lower expression at LCD and 6°C. The Aux-3 was highly expressed in the Δ*litR* mutant, relative to wild type, at HCD and 12°C and at LCD and 6°C. When comparing the Δ*ainS* mutant with the wild type (tpΔ*ainS*/WT), only the expression of *vasR1* and *vasQ1* genes of the T6SS1 cluster were significantly reduced, whereas no differences in T6SS2 and T6SS3 gene clusters were observed. The *vgrG1* of Aux-1 showed lower expression (FC value = −2.07) at LCD and 6°C while the *hcp1* of Aux-2 showed significant lower expression (FC < −2.0) at all conditions except at LCD and 12°C. Additionally, the genes AWOD_II_1031 and *hcp3* of Aux-3 showed significant higher expression at HCD and 12°C. Other genes of auxiliary clusters in Δ*ainS* mutant did not show significant differential expression.

### Potential T6SE Molecules Identified in *A. wodanis* 06/09/139

We predicted 80 potential effectors proteins using the T6SE prediction tool Bastion 6 (Supplementary Table 7) (91). Of the 80 potential effectors, 29 could be annotated, while 51 were identified as putative proteins. Of the potential effectors, 44 were located on chromosome 1, 33 effectors on chromosome 2 and 2 effectors were predicted to be located on plasmid p20. Among the annotated T6SEs, we found potential cell wall degrading effectors such as N-acetylmuramoyl-L-alanine amidase and hydrolases, membrane degrading effectors including phospholipases, and several nucleotide degrading nucleases. Only a few effectors such as a porin-like protein H (AWOD_I_1000), a RHS protein (AWOD_II_0133) and type VI secretion system secreted protein Hcp (AWOD_II_1032) were located close to the main T6SS clusters, whereas the rest were located in different locations on both chromosomes.

### Differential Expression of T6SEs in *A. wodanis*

In wild type, 24 effector genes were differentially expressed at HCD (tpHCD/LCD) at 6°C, while 19 effectors were differentially expressed at 12°C (Supplementary Table 7). These effector genes encoded several proteins including outer membrane proteins, membrane proteins, proteins with PAAR motif, lipoproteins with LysM domain, a sulfite reductase [NADPH] flavoprotein alpha-component, a secreted endonuclease I and a choline dehydrogenase. Interestingly, in the wild type at HCD and 6°C, an FC value of 173.29 was found for choline dehydrogenase (AWOD_II_1235), which was 20 times higher than at 12°C (FC = 8.99). When the wild type profiles at high and low temperatures were compared (tp12°C/6°C), 18 effectors and 22 effectors were differentially expressed at HCD and LCD, respectively. These effectors comprised putative exported protein, putative lipoprotein, putative beta-lactamase, amine oxidase and choline dehydrogenase.

In the Δ*litR* mutant, genes encoding effector molecules with significantly lower expression (FC < −2.0) were putative exported proteins (AWOD_I_0175, AWOD_I_1184, AWOD_II_0501, and AWOD_II_0656), outer membrane protein (AWOD_I_1120), putative lipoprotein (AWOD_I_1186, AWOD_II_0440, AWOD_II_0804, and AWOD_II_1206), putative polysaccharide deacetylase (AWOD_I_1338), membrane protein (AWOD_I_1567), amine oxidase (AWOD_II_0852) and phospholipase (AWOD_II_1212). The effector molecules genes differentially expressed in Δ*ainS* mutants included a putative histidine decarboxylase (AWOD_I_1509), and a secreted endonuclease (AWOD_II_0252). Additionally, genes encoding effectors such as a porin-like protein H (AWOD_I_1000), an endonuclease I precursor (AWOD_I_2248) and a 6-phospho-beta-glucosidase (AWOD_I_0029) were differentially expressed in both Δ*litR* and Δ*ainS* mutants. The differential expression of effector molecules in Δ*litR* mutant was seen more often during LCD and 6°C than at other experimental conditions like HCD and 12°C (Supplementary Table 7).

## Discussion

*A. wodanis* is frequently isolated together with *M. viscosa* during the winter ulcer outbreaks and believed to be involved in the progression of winter ulcer disease (7, 73, 74, 78). Although *M. viscosa* is the main agent causing the disease, the reason for its co-existence with *A. wodanis* is not yet clear. Bacteria use several strategies to compete for niche adaptions, which are known to be regulated by various mechanisms, including QS (49, 100–102). In our previous study we have shown that QS in *A. wodanis* regulates various phenotypic traits and cytotoxicity on CHSE cell line (80). In this study, we performed genome analysis and gene expression profiling of *A. wodanis* to explore the QS system and its role in regulating the survival strategies.

### Total DEGs

When comparing the transcriptome profile of the *A. wodanis* wild type between cell densities (tpHCD/LCD) and temperatures (tp12°C/6°C), the strongest effect in terms of number of DEGs were at LCD (164 more genes than at HCD) and at 6°C (140 more genes than at 12°C). Furthermore, the functional mapping of DEGs revealed that families such as *secretion system, prokaryotic defense system* and *transporters* were highly expressed at HCD compared to LCD. As expected, the gene families related to protein synthesis such as *ribosomes, ribosome biogenesis* and *transfer RNA biogenesis* were less expressed at HCD compared to LCD (tpHCD/LCD). Similar cell density-dependent gene expression has been reported in *Aliivibrio salmonicida*, where about 1,000 genes were differentially expressed in response to increase in cell density (103). In the Δ*litR* mutant, 1.8 times more DEGs were observed at tpHCD/LCD at 6°C than for the wild type grown at the same condition, suggesting LitR is an important regulator in *A. wodanis* at low temperature. Furthermore, in *A. wodanis*, the lowly expressed genes in comparison tp12°C/6°C were related to protein synthesis such as *non-coding RNAs, ribosomes, transfer RNA biogenesis* and *ribosome biogenesis*. This suggests that genes related to protein synthesis are less expressed at 12°C compared to 6°C. Temperature is one of the major environmental stress factors that bacteria encounter in nature and many genes respond to temperature changes. In addition, the functional families such as the *secretion system* and *prokaryotic defense system* were also regulated by temperature in *A. wodanis*. For example, in *V*. *parahaemolyticus* 13% of DEGs were observed when the bacteria were grown at 15°C and 42°C compared to its optimal temperature 37°C, where genes related to energy metabolism were highly affected due to temperature change (104). In *A. wodanis*, more DEGs were identified in tpΔ*litR*/WT (LCD) and tpΔ*litR*/WT (6°C) compared to at HCD and 12°C, suggesting LitR is a global regulator when the temperature is low and the cells are in the early log phase. This is in contrast to *A. salmonicida*, where the numbers of DEGs in Δ*litR* mutant were higher at HCD compared to LCD at 12°C (103). The comparison between the transcriptome profiles of mutants and wildtype (tpΔ*litR*/WT and tpΔ*ainS*/WT) showed about 5 to 24 times more DEGs in tpΔ*litR*/WT compared to tpΔ*ainS*/WT at both cell densities and temperatures. This indicates that only a few genes seem to be regulated through the AinS-dependent QS system. This is similar to *A. salmonicida*, where only about 20 genes were differentially expressed in Δ*ainS* mutant when compared to wild type, whereas in *litR* mutant compared to wild type, 3 to 10 times more DEGs were found (103, 105). Moreover, in this study, the transcriptomics was performed at OD_600_ of 6.0, which is a late log phase in *A. wodanis* while it can reach an OD_600_ of ~8.0. Therefore, the AHL-mediated gene regulation in *A. wodanis* may differ at OD_600_ higher than 6.0. The functional mapping of DEGs from comparisons tpΔ*litR*/WT and tpΔ*ainS*/WT showed that the genes affected by LitR and AinS in *A. wodanis* mainly belongs to the families such as *secretion system* and *prokaryotic defense mechanism*, which contained the T6SS and CRISPR genes, respectively. Nevertheless, other families such as *enzymes, bacterial motility proteins* and *transporters* were also found to be affected by LitR and AinS in this study. LitR and AinS in *A. wodanis* also influenced various pathways such as metabolic pathways, *Quorum sensing*, and *two-component systems, suggesting* their role in signaling mechanisms and metabolic activities. QS regulation of metabolic functions such as glucose uptake, phosphoenolpyruvate-dependent sugar and nucleotide biosynthesis are known to enhance the co-operative behavior of bacteria to survive under limited nutrient conditions (101). Similarly, the LitR and AinS regulation of metabolic pathways in *A. wodanis* may play a role in co-operative behavior under limited nutrient conditions.

### QS System in *A. wodanis*

**Figure 4:**
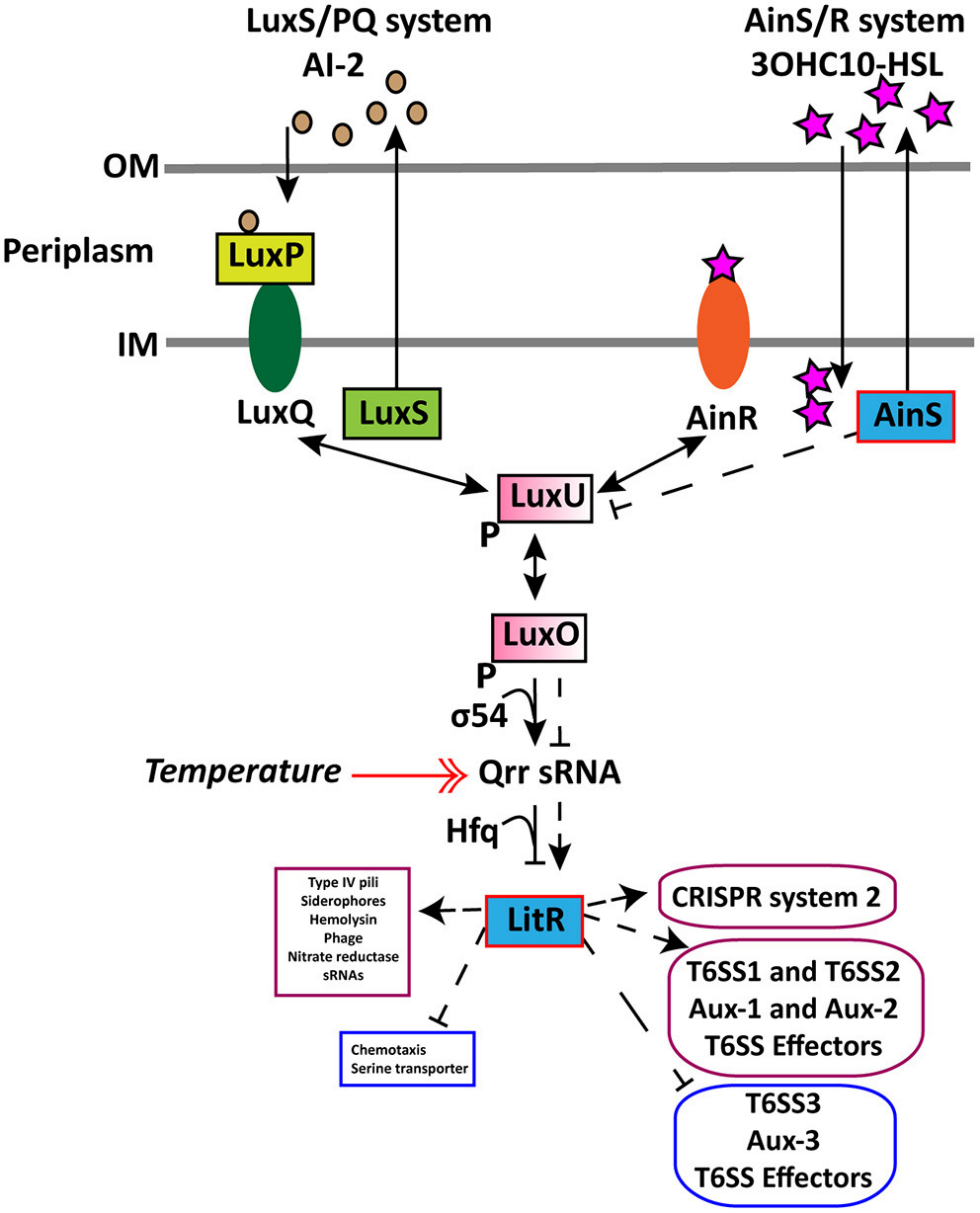
Proposed model for regulation of CRISPR and T6SS in *A. wodanis* 06/09/139 at 6°C. The model includes the two QS systems LuxS/PQ and AinS/R in *A. wodanis*. At low cell density (LCD), when the AIs concentration is low, the receptors (AinR and LuxPQ) may act as kinases and relay phosphate to LuxO via LuxU, which activates the expression of *qrr* sRNA. The Qrr sRNA inhibits the expression of *litR*. When the AI concentration is high at high cell density (HCD), they bind to the receptors to dephosphorylate LuxO and inactivate *qrr* sRNA. Inactivation of *qrr* sRNA, in turn, activates *litR*, which regulates many genes. Arrowheads at both ends indicate phosphorylation relay, and the symbol “P” indicates the phosphorylated state. AinS seems to affect luxU negatively and the *litR* expression positively. LitR seems to positively affect the genes involved in T6SS1, T6SS2, Aux-1, Aux-2, T6SS effectors and CRISPR system 2 and, conversely, affect T6SS3, Aux-3 and T6SS effectors negatively. Thin line and dashed line with arrowhead indicate regulation at LCD and HCD, respectively, where arrowheads indicate increased expression (+) and bars indicate decreased expression (-). A double arrowhead red line indicates the increased expression of *qrr* sRNA at 12°C compared to 6°C. Genes with significantly higher and lower expression are presented in purple and blue boxes, respectively.

In wild type, we found that an increase in cell density resulted in a higher expression of the AHL autoinducer synthase *ainS* and the master QS regulator *litR* when grown at 6°C and not at 12°C. This demonstrates that cell density only affects the expression of *litR* and *ainS* genes at low temperature. Similar increased expression of *ainS* was also observed in the Δ*litR* mutant strain when comparing HCD to LCD (tpHCD/LCD), indicating that the *ainS* expression is not entirely LitR-dependent. These results confirm our previous work, where we showed that the 3OHC10-HSL production is cell density- and temperature-dependent (80). Similar to AinS in *A. salmonicida* (105), the AinS in *A. wodanis* negatively affected the *luxU* expression. This suggests that the decreased expression of *luxU* could repress *qrr* and activate *litR* in *A. wodanis*. Similarly, in this study, *luxU* was affected by AinS only at 6°C, which could suggest that low temperature affects the phosphorelay function of *luxU*. On the other hand, the *qrr* gene was more expressed at 12°C compared to 6°C. Qrr sRNAs in vibrios are known to regulate several target mRNAs, including the master QS regulator LuxR (106). In *A. fischeri*, Qrr sRNAs negatively regulates the production of LitR (16). Therefore, the increased expression of *qrr* in *A. wodanis* at 12°C may negatively affect the production of LitR and, subsequently, the QS dependent gene expression. Our results illustrate that *litR, ainS, luxU, qrr* sRNA in the *A. wodanis* QS system were differentially expressed in a cell density and temperature-dependent manner. The proposed QS model in *A. wodanis* and its regulation of target genes are presented in Figure 4.

In this study, LitR in *A. wodanis* did not affect the *ainS* expression, except at LCD and 12°C (FC = −1.93) in the Δ*litR* mutant. This confirms our previous work where we showed that the 3OHC10-HSL production is not completely controlled through LitR (80). However, LitR in *A. fischeri* and *A. salmonicida* has been shown to significantly upregulate AinS (11, 107). In *A. fischeri*, several LitR-independent regulatory mechanisms have been reported to regulate *ainS*, such as *ainS* auto-regulation or cyclic AMP receptor protein- and glucose-mediated mechanisms (11, 108). Similarly, in *A. wodanis*, other regulatory mechanisms may regulate the expression of *ainS*. The deletion of *ainS* in *A. wodanis* negatively affected the *litR* expression. In the Δ*ainS* mutants, the FC values of *litR* were slightly higher at HCD compared to LCD at both temperatures, which suggests that 3OHC10-HSL partly activates *litR* independently of the temperature. Similarly, the autoinducer synthase C8-HSL in *A. fischeri* positively affected *litR* in a cell density-dependent manner (14).

### Regulation of CRISPRs and Spacers

*A. wodanis* harbors a type I-F CRISPR system and a CRISPR system without *cas* loci, the CRISPR system 1 and CRISPR system 2, respectively. CRISPR systems without *cas* loci are known as orphan arrays, where most of these are known to be non-functional (39). However, some of the orphan CRISPR arrays may function together with invading *cas* genes or the *cas* genes present in a different location within the same genome (109). In *A. wodanis*, the CRISPR system 1 consists of 25 spacers, whereas CRISPR system 2 consists of 40 spacers. The difference in the number of spacers in *A. wodanis* suggests that the CRISPR system 1 is less active than the CRISPR system 2 or is a remnant of a previously active CRISPR system. In addition, the spacers were not identical between CRISPR systems 1 and 2, indicating both are working independently of each other or that CRISPR system 2 has been introduced later than CRISPR system 1. The *cas* genes in *A. wodanis* shows ~90% amino acid similarity to the *cas* genes in *M. viscosa*, suggesting that the CRISPR system 2 has been horizontally transferred from *M. viscosa*. We speculate here that both bacteria can defend themselves against the same agents, which may favor their co-existence during the development of the winter ulcer disease. Similarity search of the spacers in *A. wodanis* against the phage databases revealed *Vibrio* phage CTX plasmid pCTX-2 and *Staphylococcus* phage phiSP38-1.

The expressions of *cas* genes (*cas1, cas3*, and *csy3*) in wild type seems to be cell density-dependent at 6°C, where the *cas* genes showed increased expression at HCD compared to LCD. However, cell density had no significant effect on the expression of CRISPR arrays of CRISPR systems 1 and 2 regardless of temperatures. Therefore, the CRISPR systems are partially dependent on cell density. Previous studies suggest that bacteria are at higher phage risk at higher cell density densities, and thus protection against phage is important at HCD (45). This suggests that *A. wodanis* positively influences *cas* genes as the cell density increases, and this might provide protection upon phage infection at HCD.

The CRISPR arrays of CRISPR system 1 in *A. wodanis*, was more highly expressed at 6°C when compared to 12°C, suggesting that the temperature 6°C plays a significant role in CRISPR array 1 expression. Temperature is known to regulate CRISPR-Cas genes in *P. aeruginosa* (45, 102). However, temperature difference did not regulate the expression of neither the *cas* operon nor the CRISPR array of the CRISPR system 2.

The complete *cas* operon of CRISPR system 2 in *A. wodanis* showed decreased expression in the Δ*litR* mutant irrespective of cell densities and temperatures, which demonstrates that the CRISPR system 2 is regulated through LitR. In the Δ*litR* mutant, the expression of the *cas* genes was higher at 6°C compared to 12°C at HCD. At LCD, only the expression values of the *cas1* and *cas3* were higher at 6°C compared to 12°C, while other genes such as *csy1234* were higher at 12°C. Although LitR in *A. wodanis* activates the CRISPR system 2, the QS regulation is not completely cell density and temperature-dependent. Furthermore, the data shows that the CRISPR system 1 was more highly expressed in Δ*litR* mutant compared to wild type, implying that LitR negatively affects CRISPR system 1.

In the Δ*ainS* mutant, although a lower expression was observed on genes *csy2, csy3*, and *csy4*, the expression of CRISPR arrays of CRISPR system 2 and CRISPR system 1 were not changed by inactivation of *ainS*. Hence, the activation of the CRISPR system 2 in *A. wodanis* seems to be partly dependent on the AHL-mediated QS system. The QS activation of the CRISPR system has been reported as an efficient mechanism to enhance the benefit-to-cost ratio in *P. aeruginosa* (44, 45). Although several vibrios and aliivibrios comprise both QS and CRISPR systems, the QS regulation of the CRISPR system has not been described yet. Expression of the CRISPR system can be costly to the bacteria, and in some bacteria like *Streptococcus thermophilus*, the CRISPR system is constitutively expressed and confers a fitness cost (110). Therefore, the CRISPR system may only be expressed during phage infection or due to environmental cues (111–113). Similarly, the cell density, temperature and QS dependent regulation of CRISPR may enforce a benefit for *A. wodanis* during the development of winter ulcer.

Despite exhibiting CRISPR systems, one intact prophage was identified in *A. wodanis* chromosome 1 and several incomplete prophages in both chromosomes and the plasmids. Phages that infect *P. aeruginosa* are known to escape the type I-F and I-E CRISPR systems using anti-CRISPR proteins (114, 115). Therefore, we searched for anti-CRISPR proteins in *A. wodanis* using ArcFinder (116). No anti-CRISPR proteins were predicted, suggesting no self-targeted protospacers that prevent CRISPR-Cas response in *A. wodanis*. Although defective in their function, these incomplete phages are known to have adaptive functions in their hosts (117). Several of these incomplete phages are a putative source for phage-derived molecular products such as gene transfer agents, bacteriocins, phage killer particles, and they can also interfere with assembly of other phages (117, 118). The hypothetical proteins of intact and incomplete prophages from PHASTER output were searched against non-redundant protein database using BLASTP with default parameters. The hypothetical proteins present in the intact prophage were annotated as structural phage-related proteins. However, the incomplete prophages encoded several conserved proteins (with ≥ 90% amino acid identity and query coverage) such as type I restriction endonuclease subunit M, ClbS/DfsB family four-helix bundle protein, DUF559 domain-containing protein, type II toxin-antitoxin system RelE/ParE family toxin and other conserved hypothetical proteins (Supplementary Table 8). These proteins encoded by the incomplete prophage may play a role in adaptive functions of *A. wodanis*. However, further research is required to verify this.

### Regulation of T6SSs, Aux, and T6SE Molecules

Bacteria secrete many virulence factors during the host-pathogen interface, not only to overcome the host's immune system but also for inter-bacterial competition (119). T6SS is an important virulence and survival factor present in about a quarter of known Gram-negative bacteria (120). *A. wodanis* genome revealed three T6SSs and four auxiliary clusters. Many bacteria possess more than one T6SS, which likely have different functions. For example, *Burkholderia thailandensis* possesses five T6SSs copies with different functions, where T6SS1 enhances the growth in the presence of other competing bacteria and T6SS4 is involved in the manganese transport to survive under oxidative stress, and the T6SS5 is involved in virulence in the murine model of pneumonic melioidosis (121, 122). Similarly, the multiple T6SSs in *A. wodanis* may have different cellular functions.

The T6SS1 in *A. wodanis* is highly similar (71–88%) to the T6SS system 1 in *A. fischeri* MJ11, while the T6SS2 shows high similarity (54–92%) to *V. cholerae* O1E1 T6SS and *V. fluvialis* T6SS2 (90, 123, 124). The T6SS3 in *A. wodanis* shows similarity (33–84%) to *M. viscosa, V. anguillarum, A. salmonicida* and *Vibrio tapetis* (48, 125, 126). Hcp and VgrG are essential components for the proper functioning of T6SS (127). *A. wodanis* encodes *vgrG1* in Aux-1, however, it contains *hcp* in neither T6SS1 nor Aux-1 clusters. In *A. fischeri* MJ11, the T6SS1, which is similar to the T6SS1 of *A. wodanis*, is believed to interact with eukaryotic cells and is not involved in inter-strain killing (124). Moreover, in *A. wodanis* Aux-1 is located close to the heme uptake and utilization related genes. Metals such as iron are important for many cellular processes, and the genes located close to T6SS genes are known to be involved in iron uptake, for example, in *P. aeuroginosa* (58). Thus, the T6SS1 along with the Aux-1 may possibly be involved in iron uptake from the host or environment. Except for Hcp, the other structural T6SS proteins share low-level homology between each T6SSs. This indicates that the multiple T6SSs in *A. wodanis* is not a result of a recent duplication. Four copies of *hcp* were identified in the main and auxiliary T6SS clusters of chromosome 2. The proteins Hcp1 and Hcp4 showed 60% homology to each other, whereas Hcp3 and Hcp4 showed 100% homology to each other. Besides the structural role of Hcp, it is involved in the inter-bacterial competition, bacterial invasion, adherence, and cytotoxicity against host cells, also known to have other functions in different bacteria (127, 128). Therefore, the multiple copies of *hcp* in *A. wodanis* may have different functions.

*V. cholerae* utilizes T6SS to compete against diverse eukaryotic and prokaryotic organisms (66, 129). In *V. fluvialis*, the T6SS2 is anti-bacterial and provides a better competitive fitness in the marine environment (123). The T6SS2 in *A. wodanis*, which shows higher homology to *V. fluvialis* and *V. cholerae*, may enhance *A. wodanis* through inter-bacterial competition and virulence.

The T6SS3, unlike T6SS1 and T6SS2 in addition to structural components, contains additional genes *vtsABCD* encoding transporter proteins and does not contain *vasH*. VtsA-D plays a role in stress responses, transport function and *hcp* expression in *V. anguillarum*, and VasH is essential to drive the expression of the T6SS operon by inducing *hcp* and *vgrG* expression (48, 130). The mutation in *vasH* repressed *hcp* expression and impaired its anti-bacterial activity in other vibrios (123, 131). In *V. anguillarum*, VtsA-D proteins are involved in stress responses, however, plays no role in virulence (48). Similarly, T6SS3 in *A. wodanis* is probably involved in stress responses. After *V. anguillarum*, the T6SS similar to T6SS3 in *A. wodanis* is in *M. viscosa* (mts1). The genes encoding Hcp (AWOD_II_1028 and AWOD_II_1032), which are located close to each other, shows 71% similarity to *M. viscosa hcp*, MVIS_3030 (7). This implies that the T6SS3 in *A. wodanis* might have a similar function as mts1 of *M. viscosa* during the winter ulcer disease development.

In this study, expression of the T6SS1 and T6SS2 genes and their auxiliary clusters in *A. wodanis* are dependent on cell density. Such cell density-dependent T6SS expression has been reported in *V. parahaemolyticus* (50). Some of the genes in the T6SS2 and auxiliary clusters (Aux-2 and Aux-3) were found to be altered by temperature. The expression was found to be higher at 6°C than at 12°C. In *V. fluvialis*, the T6SS2 is regulated by temperature (123). Temperature-dependent regulation of virulent factor genes may be an essential feature for many bacteria to survive in harsh environments (132).

LitR in *A. wodanis* seemed to positively affect two T6SSs (T6SS1 and T6SS2) and negatively affect the expression of T6SS3 gene cluster. This demonstrates that QS regulation of the T6SSs in *A. wodanis* is very complex. The regulation of T6SS3 by LitR in *A. wodanis* indicates T6SS3 may play different roles than T6SS1 and T6SS2. Such reciprocal regulation has also been shown in *V. parahaemolyticus*, where the QS regulator OpaR downregulates T6SS1 and upregulates T6SS2 where T6SS1 functions as anti-bacterial and T6SS2 as anti-eukaryotic (133).

LitR in *A. wodanis* seemed to positively affect only the genes encoding the outer sheath and base-plate proteins of T6SS1, suggesting these genes are QS dependent. Interestingly, LitR was involved in activating the expression of the whole apparatus of the T6SS2 system. Moreover, LitR also repressed the entire T6SS3 gene clusters but only at LCD at 6°C. Therefore, T6SS2 is ultimately QS dependent, whereas T6SS1 and T6SS3 are not completely QS dependent. Regulation of T6SS by LuxR homologs have been described in *V. cholerae* (HapR), *V. alginolyticus* (LuxR) and *V. anguillarum* (VanT) (48, 49, 51, 134). Furthermore, we observed that LitR in *A. wodanis* is a strong activator of *hcp1* expression at 6°C than at 12°C. Hence, this possibly implies that *hcp1* of T6SS2 is may be involved in the cytotoxicity in CHSE cell line (80). Temperature has been shown to influence *hcp* expression in other bacteria such as *Yersinia pestis* and *V. parahaemolyticus* (133, 135). Similarly, in *A. wodanis*, the high expression of *hcp* at 6°C indicates that the T6SS2 could be more active at low temperature (6°C).

We identified that the genes in Aux-2 encoding ankyrin repeat-containing proteins and RHS proteins were differentially expressed in tpΔ*litR*/WT at 6°C. Ankyrin repeat proteins are known to be involved in pathogenesis by imitating and impeding host function (136). RHS proteins are toxins that are exported to the cell surface through T6SS, and it mediates anti-bacterial activity (137–139). AinS had no significant effect on the main and auxiliary clusters of T6SSs like LitR. However, AinS positively influenced the expression of the *hcp1* and *vgrG1* genes and may indicate that only these T6SS genes are dependent on AHL-mediated QS. We predicted several T6SS effectors in *A. wodanis*, including lipoprotein, nucleases, membrane proteins, amidases and succinylglutamate desuccinylase. However, most of them are putative and hypothetical proteins, which require further research to confirm. From the transcriptomics data, we found that cell density regulated several predicted T6SEs in wild type. Choline dehydrogenase, an osmoprotectant enzyme that protects bacteria from adverse temperatures and other stresses, was more highly expressed at 6°C (140, 141). The temperature has affected expression of several T6SEs indicating a temperature-dependent production of effector proteins. Similar to the LitR regulation of T6SS main and auxiliary clusters, it also controlled several T6SEs. Few T6SEs were also found to be affected by AinS. This may indicate that several effectors are dependent on QS, where LitR influenced the expression higher at LCD and 6°C. Some of the known effectors included porin-like protein H (AWOD_I_1000), a molecular filter for hydrophilic compounds and bacteriocin (AWOD_p920_0063), which is a virulent factor in *A. wodanis* that modulates the growth and virulence of *M. viscosa* (7, 17). Genes encoding T6SS immunity proteins are usually located close to the genes encoding effector proteins (63). The potential immunity protein of Aux-1 (AWOD_I_1437) in *A. wodanis* shows 30% amino acid similarity to the immunity protein in *V. cholerae* strain O1E1 (90), while in Aux-2, the immunity protein (AWOD_II_0134) shows 29% amino acid similarity to *Mucilaginibacter gotjawali* (142). The immunity protein (AWOD_II_1056) in Aux-4 shows 77% amino acid similarity to a hypothetical protein in *A. fischeri* MJ11 (124).

## Conclusion

In this present study we show that cell densities and temperatures influenced the expression of many genes in *A. wodanis*. Moreover, the QS related genes in *A. wodanis* are cell density- and temperature-dependent, where 6°C plays an essential role in activating the AHL-mediated QS system. *A. wodanis* harbors two CRISPR systems, three T6SSs and four auxiliary T6SSs in its genome. We show that the CRISPR system 2 and T6SS3 of *A. wodanis* are similar to those in *M. viscosa*, the bacteria with which *A. wodanis* co-exists during winter ulcer disease. The low-temperature 6°C at which winter ulcer occurs exerts a significant effect on the expression pattern of *A. wodanis* than at high-temperature 12°C. We demonstrate here that LitR regulates CRISPR-Cas and T6SSs in a cell density- and temperature manner. Moreover, QS is found to regulate several potential T6SEs in *A. wodanis*. Thus, the QS regulation of T6SSs and CRISPR-Cas system of *A. wodanis* could be essential to understand the possible mechanisms used by *A. wodanis* during its co-existence with other bacteria like *M. viscosa* or the host.

## Data Availability Statement

The datasets presented in this study can be found in online repositories. The names of the repository/repositories and accession number(s) can be found in the article/Supplementary Material.

## Author Contributions

AM, EH, HH, and NW conceived and designed the experiments. AM performed the experiments. AM, EH, and NW analyzed the transcriptomics data. AM and NW wrote the manuscript. All authors contributed to reviewing and proof reading the final manuscript.

## Funding

This work was funded by the Research council of Norway (#270068: ELIXIR2) and UiT The Arctic University of Tromsø. The publication charges for this article have been funded by UiT The Arctic University of Tromsø.

## Conflict of Interest

The authors declare that the research was conducted in the absence of any commercial or financial relationships that could be construed as a potential conflict of interest.

## Publisher's Note

All claims expressed in this article are solely those of the authors and do not necessarily represent those of their affiliated organizations, or those of the publisher, the editors and the reviewers. Any product that may be evaluated in this article, or claim that may be made by its manufacturer, is not guaranteed or endorsed by the publisher.

## Abbreviations

AHL: N-acyl homoserine lactone
Aux: Auxiliary
Bp: Base pair
CHSE: Chinook salmon embryo
CRISPR: Clustered Regularly Interspaced Short Palindromic Repeats
DEGs: Differentially expressed genes
FC: Fold change
HCD: High cell density
LCD: Low cell density
MGE: Mobile genetic element
Min: minutes
OD_600_: Optical density measured at 600 nm
QS: Quorum sensing
RHS: Rearrangement hotspot protein
RPKM: reads per kilobase of gene per million reads mapped
T6SE: Type VI Secretion Effector
T6SS: Type VI Secretion System
tp: Transcriptome profile.
