# Supplementary material for: Quorum Sensing Controls the CRISPR and Type VI Secretion Systems in Aliivibrio wodanis 06/09/139
Source: Front Vet Sci. 2022 Feb 8;9:799414. doi: 10.3389/fvets.2022.799414 (PMC8861277; doi:10.3389/fvets.2022.799414)
Supplement: Supplementary file 4 [file Table_4.docx]

**Spacers in CRISPR array 1 and 2**

>spacer1

TAACAGTAAGCAAACGTGGATCTTTTCCTTTC

>spacer2

TTGGTATTATTATTCGTGAATTTCCGCAAAAT

>spacer3

CGAGGGGACAGAGATCCCGCCTGAATGCAGAC

>spacer4

CTTGAGCAACTTGGATTTCTTTTGATTTGTCA

>spacer5

GCCGACAAAGCTATTAAATCCATGTTTATGAT

>spacer6

TGTTTGTTCATTCTATTTCACCATTTTAATTA

>spacer7

GAAAATCCAAGGGACAGCCTACGTATTTTTTA

>spacer8

AGCGACTTGATGGTGTAGCATTAAATCCATTA

>spacer9

AATCAGCGTAATCGACTGGAAAAGTGTCTACT

>spacer10

AATCTGTGTTGATGAGTGTCATCGCTTGGATT

>spacer11

GTTGGTCTGGTTTACCCCCGTAATACTAGACG

>spacer12

CGCCGAAGTTCAACACGTCGTCGCAGAAGAAA

>spacer13

TTCTGTATGAGCAATTAAAATGTTATCGCAAG

>spacer14

GCGGAGGTGTTGGTGGGTCAAACGGTTCAATT

>spacer15

TTGTTATGACTTTCCGACCTTTCTCCAAAGCA

>spacer16

GGAAGTGAATTAGAACTCGCGTTCCTTCTTGT

>spacer17

ATACAGTCGGAGAAGTTAAGATTCTTGCGTAT

>spacer18

TAATAATGTTTTTTGTTGGATCTTCTGGCAAT

>spacer19

TGATTGGTCTTGCTGCGATTGGTTTCGGTATC

>spacer20

ATGACACTGTTCACTTTCAGATTAATGGGACT

>spacer21

TTTGGTTTGAATTACTTCCTGCTTTGCGTGAG

>spacer22

TTTGTCGCCAGTTTTAGATCCTTTTTGACAGG

>spacer23

GTACTGCATCAATGACACCCGAATCATCATAA

>spacer24

GGTAATCCCTCACGTTTCATAGCGTCAAATAAT

>spacer25

TGAGCAAGACTAACACCAGCTAAATCAGCAGA

**CRISPR array 2**

>spacer1

ACCATGGAATATTTGCGGCGTTTGCCTGGGGC

>spacer2

TTGTGTTTATATTTACACCATCAATAACGAAA

>spacer3

AGCTATACCTTTAACAGCTAAGGTGTGGTGTG

>spacer4

ATTTTTTGAATACCATTTACCCGGGCGGTGTT

>spacer5

CTCTGTTATTTACTATAACGGTTCGAATAACT

>spacer6

TAGAAGATATTGAAACCAATACACTTAAACTA

>spacer7

ATCACTAAGAATGTGGGTCTTGCTGCTTCTGC

>spacer8

ATGTGGTCTAGCTTAGCTAGTCTTACTATGGA

>spacer9

TTCAATTACTTGAGTAGCCATACTGATACAGT

>spacer10

CTTATCACTCTTCGTCAAATTTAAGGCATTCA

>spacer11

AGCAAATGAGATTACGCGCTCTTTTGCTAATT

>spacer12

CTCTTTTAACTCTTTAGTTTGCTGCTTGATTT

>spacer13

CGATAAACTATAAAGAAAGTTTTAAATGTAAA

>spacer14

ACCTAGCCGCTCGAATGCTAATGACTCCCACA

>spacer15

ATGATCGCAGTTGCTAGAGGTAAGCGTAACGA

>spacer16

CATCATAAGGCTGTCCTGGAGTTTGAGACGGT

>spacer17

AGGAGGTCGATTTAACAGATGATTGATGAACT

>spacer18

TTATATGATAGCTATGGTCTGGTGAGATAAGA

>spacer19

CTCAACTTGCGTTTTCTCATCAACAAAACAAA

>spacer20

ATTTAGCGGTTGCCTGTTTTGCTTGAGCAAATT

>spacer21

AAACTATCTACGATTGGAACGATTGCCGCTACG

>spacer22

GAAACTTTAATTTTTTCTTGCTTGGATGAACT

>spacer23

TATTTAACTTCAATACCGCCAAACGTCAGTTG

>spacer24

ATGCAACAGCTTCATAGCTTTTTCCGCCACCA

>spacer25

AAATGAATAAAGAACTTTTAAGCGTAATTAAA

>spacer26

TATTCAACGGTCAAGTTTCCCCCGCTTTTGATG

>spacer27

AAGAAATCATAACATCATCACAAACTTGAGTAA

>spacer28

TTGCAAATGAAAATTTCGTAGTGGTTTCGTCC

>spacer29

AACCATCCGCAATCTCTCGACAGGTCTCTGGA

>spacer30

AATAGATTGAATAATTCGTGAATCATCAAATG

>spacer31

ATATAGAGGGATCATTAACTGGAATAGAAAAC

>spacer32

GGTTCTGTGGCTTTGGGTATTCCTCAAGCTAT

>spacer33

TATTAATCATATTATGAACCCAATCTAGTAAA

>spacer34

CTCAAAAGCTACTGCACACCAGTTACACTGTC

>spacer35

TGTAACGCCCCCACACTATCCATAATTTAATA

>spacer36

GATGAAAAAGGCGCAAAGAAGTACCGTTTTAA

>spacer37

ATTGCTCCTGATGGCTCTAAATGGGCTTGTGT

>spacer38

TTGTTCCTATTGACGGTTATATTGCTCCTGAT

>spacer39

GCACCTAGGGTTATTTTAGGATTATACTTATT

>spacer40

TTGATAAGGGTTTTTTAACTAAACGTTCTGGG
