# Supplementary material for: Quorum Sensing Controls the CRISPR and Type VI Secretion Systems in Aliivibrio wodanis 06/09/139
Source: Front Vet Sci. 2022 Feb 8;9:799414. doi: 10.3389/fvets.2022.799414 (PMC8861277; doi:10.3389/fvets.2022.799414)
Supplement: Supplementary file 9 [file Image_1.pdf]

A)

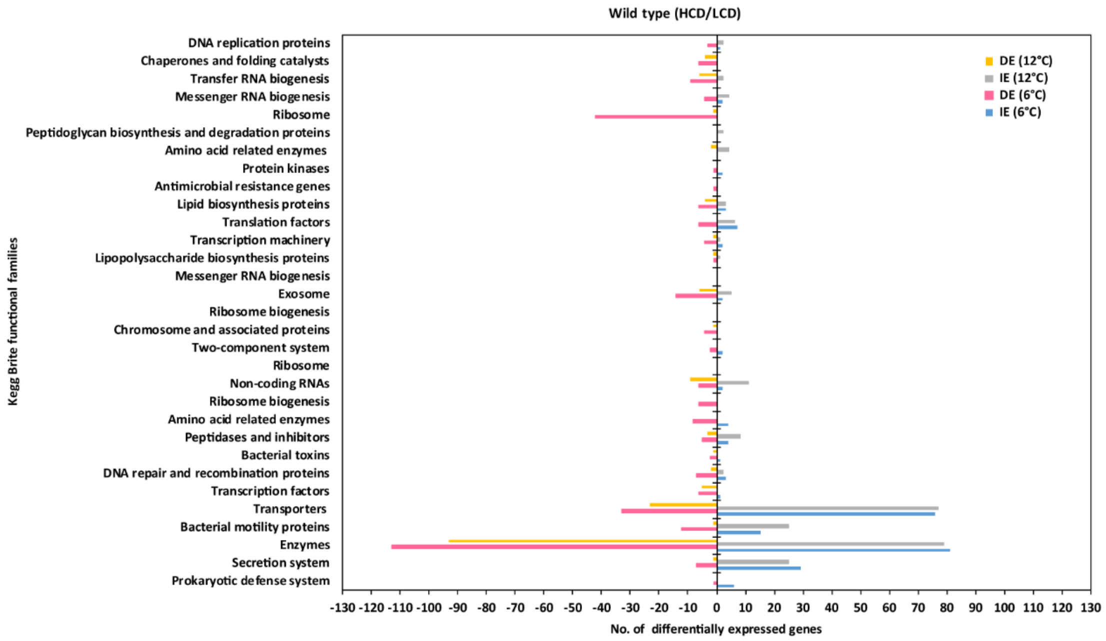

B)

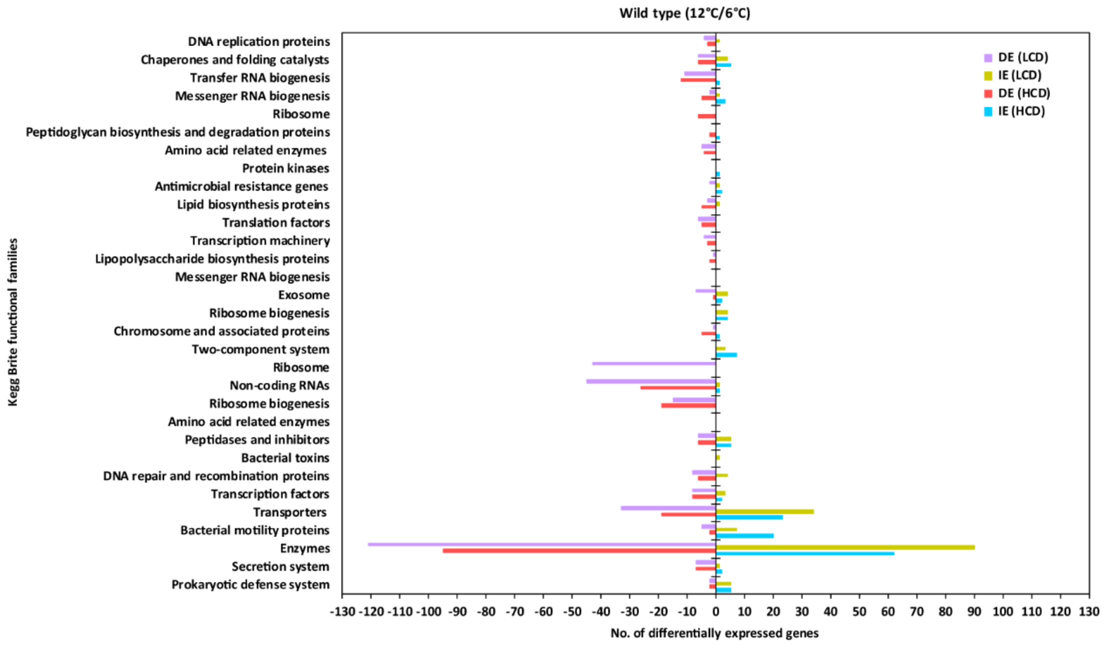

Figure S1. Functional gene family mapping of DEGs in wild type. (A) and **(B)** Bar chart showing the increased and decreased expression of genes in wild type compared between cell densities (HCD/LCD) and temperatures (12°C/6°C) sorted into different functional families respectively. IE and DE indicate increased and decreased expression respectively.
