# Supplementary material for: Quorum Sensing Controls the CRISPR and Type VI Secretion Systems in Aliivibrio wodanis 06/09/139
Source: Front Vet Sci. 2022 Feb 8;9:799414. doi: 10.3389/fvets.2022.799414 (PMC8861277; doi:10.3389/fvets.2022.799414)
Supplement: Supplementary file 10 [file Image_2.pdf]

A)

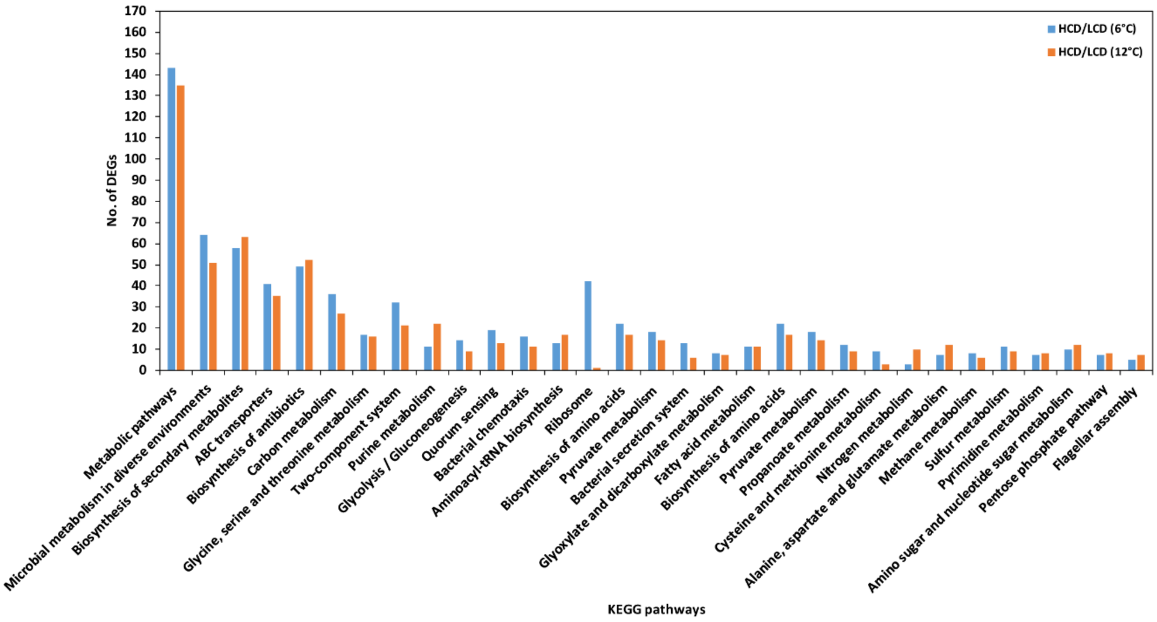

B)

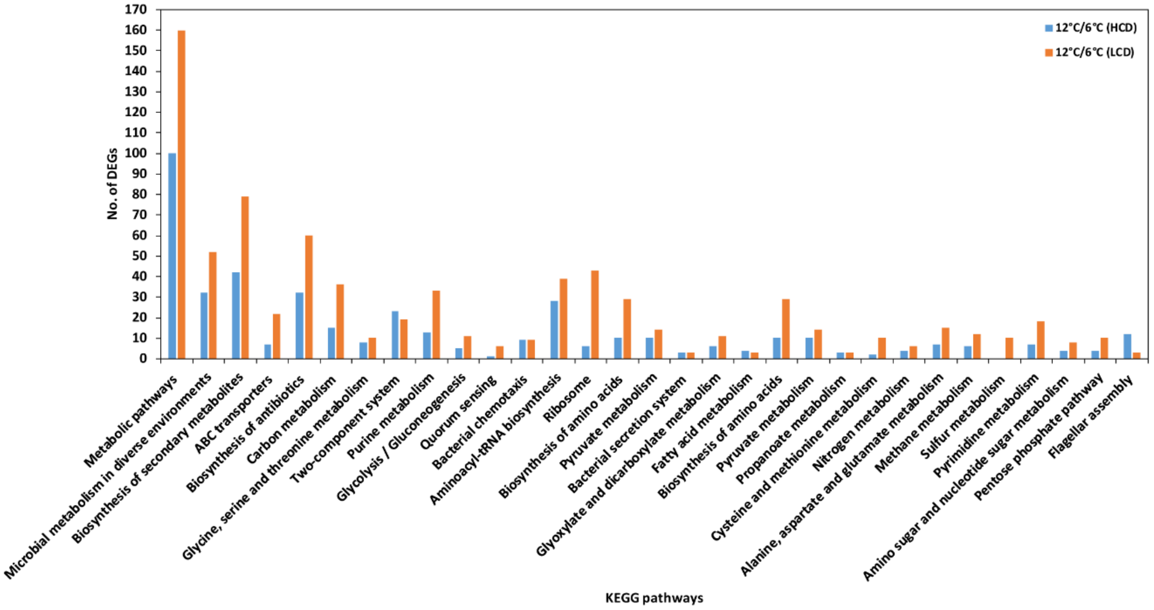

Figure S2. KEGG pathway mapping of DEGs in wild type. (A) and **(B)** Bar chart showing DEGs in comparisons WT (HCD/LCD) and WT (12°C/6°C) mapped into top 31 KEGG pathways of *A. wodanis* respectively.
