# Supplementary material for: Quorum Sensing Controls the CRISPR and Type VI Secretion Systems in Aliivibrio wodanis 06/09/139
Source: Front Vet Sci. 2022 Feb 8;9:799414. doi: 10.3389/fvets.2022.799414 (PMC8861277; doi:10.3389/fvets.2022.799414)
Supplement: Supplementary file 11 [file Image_3.pdf]

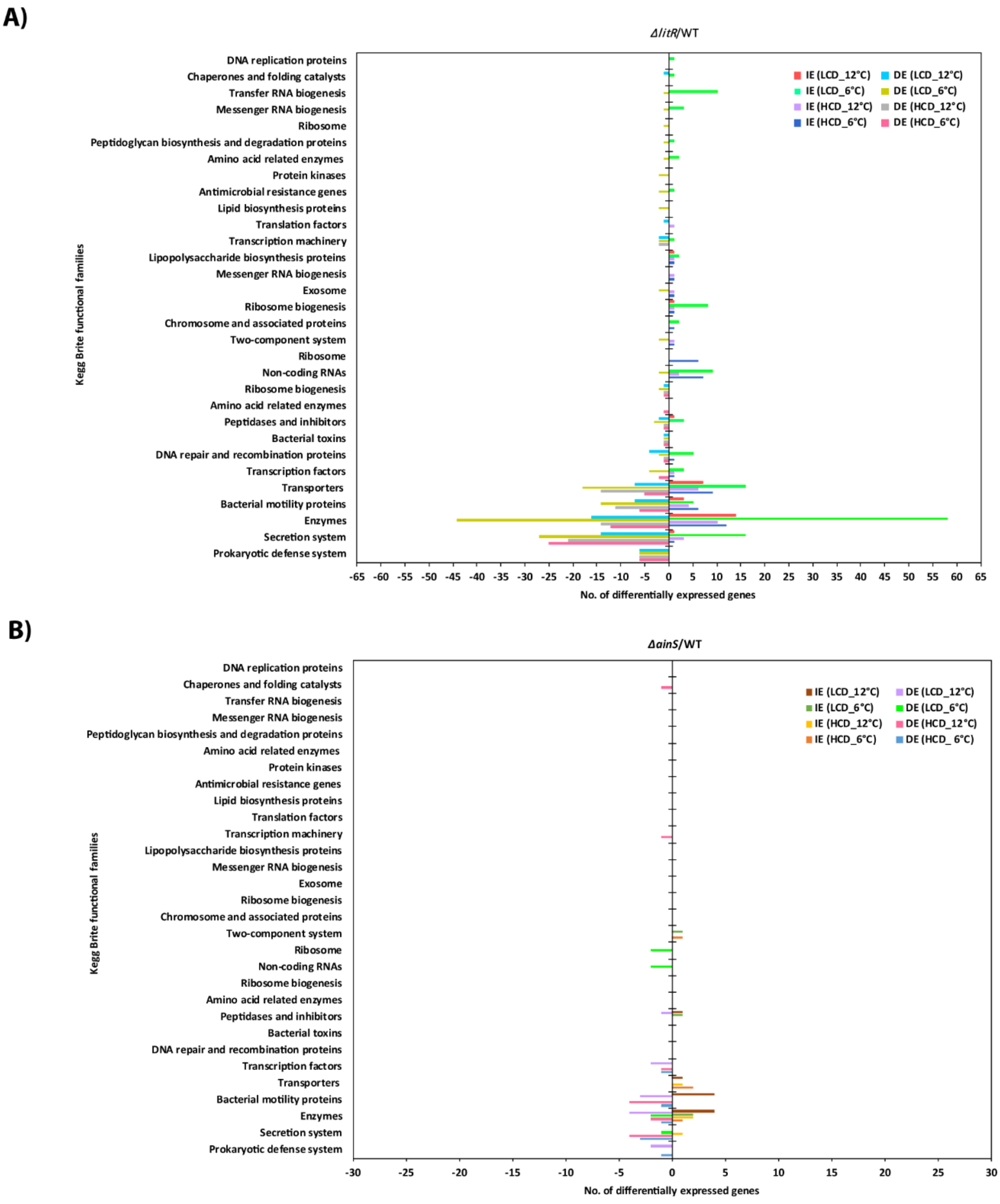

Figure S3. Functional gene family mapping of DEGs in *litR* and *ainS* mutants compared to wild type. (A) and **(B)** Bar chart showing DEGs from *ΔlitR*/WT and *ΔainS*/WT at two different cell densities and temperatures sorted into different functional families. IE and DE indicate increased and decreased expression respectively.
