# Supplementary material for: Quorum Sensing Controls the CRISPR and Type VI Secretion Systems in Aliivibrio wodanis 06/09/139
Source: Front Vet Sci. 2022 Feb 8;9:799414. doi: 10.3389/fvets.2022.799414 (PMC8861277; doi:10.3389/fvets.2022.799414)
Supplement: Supplementary file 12 [file Image_4.pdf]

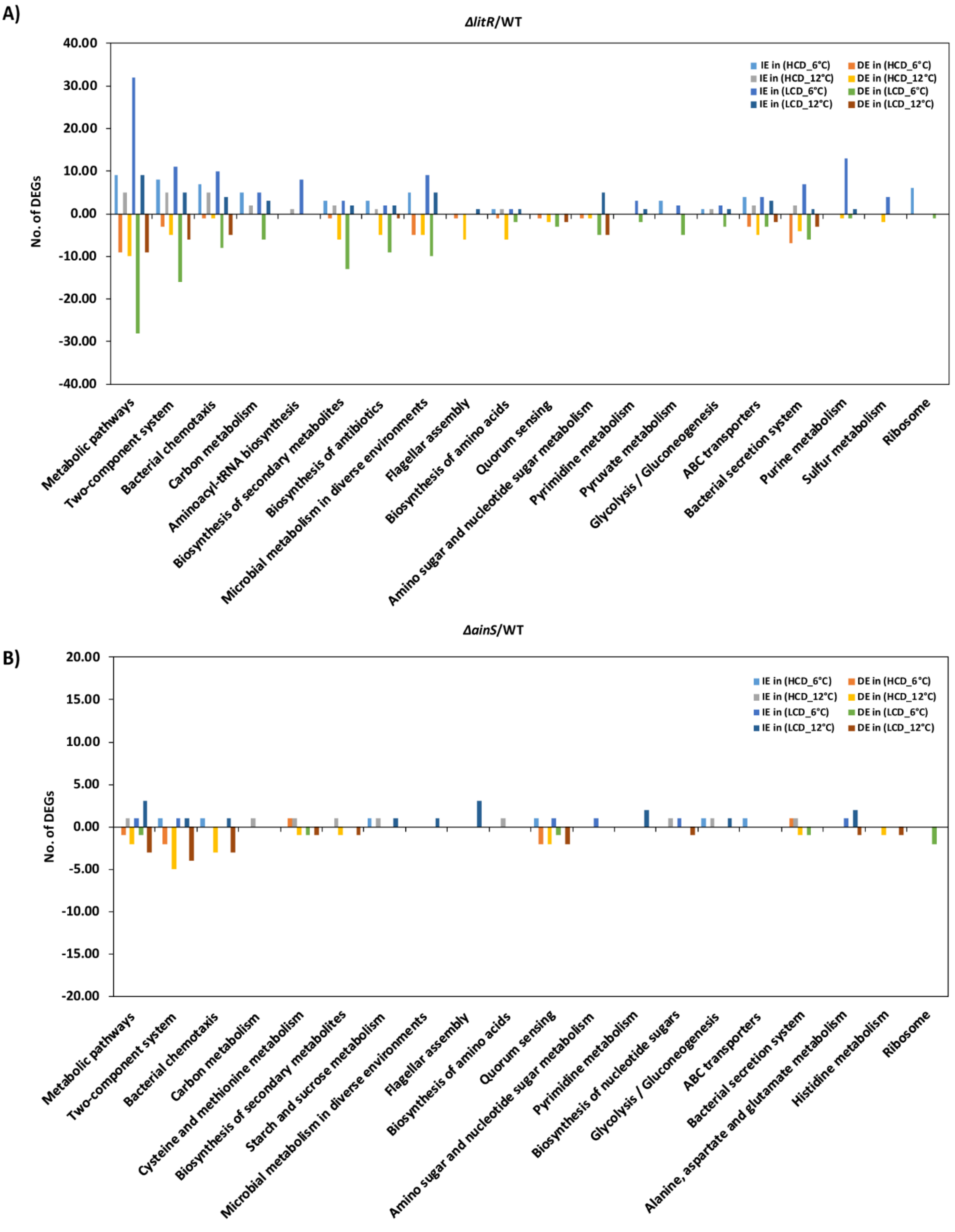

Figure S4. KEGG pathway analysis of DEGs in *litR* and *ainS* mutants compared to wild type. (A) and **(B)** Bar chart showing genes with increased and decreased expression in comparisons *ΔlitR*/WT and *ΔainS*/WT mapped into top 20 KEGG pathways of *A. wodanis* respectively. IE and DE indicate increased and decreased expression respectively.
